# Radio(chemo)therapy in Elderly Patients with Esophageal Cancer: A Feasible Treatment with an Outcome Consistent with Younger Patients

**DOI:** 10.3389/fonc.2014.00100

**Published:** 2014-05-12

**Authors:** Philippe Rochigneux, Michel Resbeut, Frédérique Rousseau, Erwan Bories, Jean-Luc Raoul, Flora Poizat, Laurence Moureau-Zabotto

**Affiliations:** ^1^Department of Radiation Oncology, Institut Paoli Calmettes, Marseille, France; ^2^Department of Medical Oncology, Centre Hospitalier Universitaire Vaudois, Lausanne, Switzerland; ^3^Department of Oncogeriatry, Institut Paoli Calmettes, Marseille, France; ^4^Endoscopic Unit, Institut Paoli Calmettes, Marseille, France; ^5^Department of Medical Oncology, Institut Paoli Calmettes, Marseille, France; ^6^Department of Biopathology, Institut Paoli Calmettes, Marseille, France

**Keywords:** esophageal neoplasm, elderly patients, radiotherapy, chemoradiotherapy, esophagectomy

## Abstract

**Background:** Although the prevalence of esophageal cancer increases in elderly patients, its clinical history and outcome after treatment remain poorly described.

**Methods:** Between January 2001 and December 2011, 58 patients (pts) older than 75 years received 3D-conformal radiotherapy (mean dose 51 Gy) in two French cancer centers. 47/58 (82%) patients received concomitant chemotherapy (with CDDP and/or FU regimens) and 8 patients underwent surgery after primary radiochemotherapy (RCT).

**Results:** Median age was 77.9 years and the performance status (PS) was 0 or 1 in 89%. Tumors were mainly adenocarcinoma of lower esophagus or gastroesophageal junction (*n* = 51, 89%), T3T4 (*n* = 54, 95%), and N1 (*n* = 44, 77%). The mean follow-up was 21.9 months. In the overall population, the median progression-free survival was 9.6 months and median overall survival (OS) was 14.5 months. Using univariate analysis, OS was significantly associated with age (*p* = 0.048), PS (*p* < 0.001), and surgery (*p* = 0.035). 35 (60.3%) and 18 patients (31%) experienced grade 1–2 or 3–4 toxicity, respectively (CTCAE v4.0).

**Conclusion:** Radiochemotherapy in elderly patients is a feasible treatment and its outcome is close to younger patient’s outcome published in the literature. Surgical resection, after comprehensive geriatric assessment, should be recommended as the standard treatment for adenocarcinoma of lower esophagus or gastroesophageal junction in elderly patients with good PS and low co-morbidity profile, as it is in younger patients.

## Introduction

Esophageal cancer (OC) is the eighth most common cancer worldwide, with 481,000 new cases (3.8% of the total) estimated in 2008, and the sixth most common cause of death from cancer with 406,000 deaths (5.4% of the total) (1). In most western countries, squamous cell carcinoma (SCC) incidence rates continue to decrease while incidence of adenocarcinoma is rocketing up (2). Furthermore, OC has a very poor survival (overall ratio of mortality to incidence of 0.88) (3). The incidence of OC in elderly patients has rapidly increased in the Western countries over the past 25 years, with a specific mortality increasing with age (4).

Recently, the field of OC management improved in several ways: (i) demonstration that the dose of 50 Gy seemed to be the better option (5), (ii) evidence from meta-analysis in favor of neoadjuvant chemotherapy or chemoradiotherapy (6), and (iii) the demonstration of the good efficacy – safety balance in favor of FOLFOX regimen (5-FU and Oxaliplatin) when compared with usual 5-FU-CDDP (Cisplatin) (7). Nevertheless, none of these trials presented sub-group data analysis focused on elderly patients. Furthermore, patients older than 75 years are often excluded from clinical trials (8, 9) and there is a lack in prospective studies in this specific population. That leads to the fact that little is known about the optimal treatment of this population.

A retrospective cohort study of 3500 patients (10) reported that older people are less referred to cancer specialist and thus, have less intensive treatment, which may partially explain the poor results of OC management among elderly patients. Particularly, a larger number of older patients eligible for esophageal resection are contra-indicated with regards to the aggressiveness of the surgery. For non-resectable OC, the strategy generally adopted is radiotherapy with or without concomitant chemotherapy. In elderly patients however, the dose of radiation and the protocols of chemotherapy is non-consensual (for example, Servagi-Vernat et al. used CDDP alone, and Anderson et al. used 5-FU and mitomycin C in association) (11, 12).

The aim of this retrospective multicentric study was to analyze the management and the outcome of 58 elderly patients treated by radiotherapy with or without concomitant chemotherapy and with or without surgery for an OC in two French centers, between January 2001 and December 2011.

## Materials and Methods

### Eligibility criteria

Inclusion criteria were as follows: histologically proven SCC or adenocarcinoma of the esophagus or gastroesophageal junction, local or loco-regional disease at diagnosis, age ≥75 years, treatment by radiotherapy (exclusive or not) between January 2001 and December 2011 in two French centers. No selection was made regarding performance status (PS), co-morbidity, or biological characteristics. Comorbidities were estimated using the Charlson score adjusted on age (13), which combines a score for 19 disease co-morbidity categories (from 1 to 6 based on the relative risk of 1-year mortality) and the patient’s age (one point per decade from 50 to 70 years old).

### Tumoral staging and evaluation of the response

All patients underwent an initial gastrointestinal fibroscopy and ultrasonography (OGUS) with biopsies and a thoraco-abdominal CT-scan. Tumors were staged using CT-scan and OGUS, according to the seventh edition of the Union for International Cancer Control and the American Joint Committee on Cancer (14). The toxicity was evaluated by a weekly clinical examination, and with a biological evaluation before every cycle of chemotherapy. All toxicities were recorded using the Common Terminology Criteria for Adverse Events v4.0 (CTCAE). The tumor response was evaluated by an OGUS and a CT-scan realized 1 and 2 months after the end of treatment, every 3 months during the first year, and then every 6 months.

### Treatments

Every treatment was debated during a tumor board including at least a surgeon, a radiation oncologist, a medical oncologist, and a radiologist. Because of the retrospective approach of this study, an onco-geriatric evaluation was not systematically realized.

Radiation therapy was delivered using 3D-conformal RT. Gross Tumor Volume (GTV) was determined with every useful information (clinical examination, CT-scan, and endoscopy) and included the primary tumor site and regional macroscopically involved lymph nodes. For patients eligible for a surgical resection, the Planning Target Volume (PTV) was defined by a proximal and distal margin of 5 cm and radial margin of 1 cm around the GTV. In case of stomach’s extension, the upper third part of the stomach was added to the PTV. For patients non-eligible for surgery, a radiation boost was often delivered on the GTV plus 2 cm margin in longitudinal extension, and 1 cm margin in sagittal and axial extension.

Concomitant chemotherapy was performed whenever possible, as decided by the multidisciplinary consultation meeting. Systemic treatment regimen was adapted to patient’s characteristics (PS, co-morbidity, and biological results) and was based on platinum derivatives and/or 5 FU. Surgery was performed in accordance with the tumor board decision, after a surgical and a pre-anesthesia evaluation, and after a neoadjuvant RCT of 45 Gy with fractions of 1.8 Gy/day, 5 days a week. The choice of transthoracic or transhiatal esophagectomy was dictated by the location of the tumor and the preference of the surgeon.

### Statistical analysis

Overall survival (OS) was calculated from the date of initial positive biopsy until the date of death or the date of last follow-up. Progression-Free Survival (PFS) was estimated from the date of endoscopy to the date of progression (loco-regional or metastatic) or the date of death or last follow-up. Loco-regional progressions were proven histologically, whereas metastatic progressions were diagnosed on CT-scan. OS and PFS curves were constructed using the Kaplan–Meier method (15). Prognostic factors for OS and PFS were obtained using the log-rank test and statistical significance was defined with a *p*-value <0.05. All prognostic factors with a *p* < 0.2 were included for a multivariate analysis using a Cox regression. Surgical and non-surgical populations were compared using a Pearson’s χ^2^ test. Analyses were performed with SPSS v16.0.

## Results

### Patient and tumor characteristics

Fifty-eight patients were enrolled in this study, from two French Cancer Centers: Institut Paoli Calmettes, Marseille (*n* = 33) and Centre de Radiothérapie St Louis, Toulon (*n* = 25). The patients >75 years represent 9% of the total of patients treated by RT for an OC in these centers. Characteristics of patients and tumors are described in Table 1. Median age was 77.8 years (range of 75–87 years). Most of the patients had a good PS: PS 0 (*n* = 12, 20%) or PS 1 (*n* = 40, 69%). Concerning co-morbidity, 37 patients (64%) had a Charlson score <5 and 21 (36%) had a Charlson score ≥5, with a median Charlson score of 4 (3–7).

**Table 1:** **Patient and tumor characteristics**.

Variable	*n* = 58	(%)
**AGE**		
75–80	42	(72.4)
80–85	13	(22.4)
>85	3	(5.2)
**SEX**		
Male	43	(74.1)
Female	15	(25.8)
**WHO PERFORMANCE STATUS**		
0	12	(20.6)
1	40	(68.9)
2	5	(8.6)
3	1	(1.7)
**CHARLSON SCORE ADJUSTED ON AGE**		
3	17	(29.3)
4	20	(34.4)
5	13	(22.4)
6	5	(8.6)
7	3	(5.2)
**LOCALIZATION ESOPHAGUS**		
Upper one-third	2	(3.4)
Middle one-third	5	(8.6)
Lower one-third	20	(34.5)
GOJ	31	(53.4)
**STAGE**		
TX NX	2	(3.4)
T1N1	1	(1.7)
T2N1	1	(1.7)
T3N0	4	(6.9)
T3N1	37	(63.8)
T3N2	1	(1.7)
T3NX	7	(12.1)
T4N0	1	(1.7)
T4N1	4	(6.9)
**HISTOLOGICAL TYPE**		
Adenocarcinoma	43	(74.1)
Squamous cell carcinoma	14	(24.1)
Undifferentiated	1	(1.7)
**HISTOLOGICAL DIFFERENTIATION**		
Well	14	(24.1)
Moderately	13	(22.4)
Poorly	7	(12.1)
Unknown	24	(41.4)

*GOJ, gastroesopageal junction*.

Among all the 58 histologically proven carcinoma, 43 were adenocarcinoma (74%). Tumors involved mostly the gastroesophageal junction (GOJ) (*n* = 31) and the lower third of the esophagus (*n* = 20). A large majority of the patients presented a locally advanced tumor T3N0-1 (*n* = 41) or T4 N0-1 (*n* = 5).

### Treatment characteristics

The main characteristics of the delivered treatments are described in Table 2. All patients underwent radiotherapy, with a mean delivered dose of 50.9 Gy (27–72 Gy). The median and mean numbers of treatment days were respectively 43 and 46 days (22–117).

**Table 2:** **Treatment characteristics**.

Variable	Total	
	*N*°	(%)
**RADIATION THERAPY (*N*** ***=*** **58)**		
Dose < 50.4 Gy	23	(39.6)
Dose = 50.4 Gy	12	(20.6)
Dose > 50.4 Gy	23	(39.6)
Mean dose (Gy)	50.9	(±8.4)
**CHEMORADIOTHERAPY PROTOCOL (*N*** ***=*** **47)**		
Weekly CDDP or Carboplatin	27	(57.4)
5-FU and platinum regimen	17	(36.2)
5-FU alone	3	(6.4)

*CDDP, Cisplatine; 5-FU, 5 Fluoro-Uracile*.

Forty-seven patients received concomitant chemotherapy: 20 had weekly Carboplatin (AUC2), 10 had Herskovic regimen (4 CDDP/5 FU chemotherapy courses with 5 FU 1000 mg/m^2^ at days 1–5 and CDDP at 75 mg/m^2^ at day 1), 7 had weekly CDDP (40 or 60 mg/m^2^), 4 had 5 FU-Carboplatin (every 21 days: 5 FU: 750 mg/m^2^ at days 1–5; Carboplatin AUC4), 3 had FOLFOX 4, 2 had capecitabine (100 mg/m/m^2^), and 1 had exclusive 5-FU (dose unknown).

Exclusive RT (without chemotherapy or surgery) was performed in 11 patients, because of age (*n* = 3), co-morbidity (*n* = 2), asthenia (*n* = 2), patient’s refusal (*n* = 1), or unknown reasons (*n* = 3).

Eight patients underwent esophagectomy, including seven transhiatal surgeries and one transthoracic Lewis Santy procedure. All the operated patients had GOJ cancer, an age <80 years, a Charlson score ≤4, and a good PS (PS 0–1: *n* = 7; PS 2: *n* = 1). Three patients had pathological lymph nodes on the post-operative findings (see Table S1 in Supplementary Material) but none of them was treated with an adjuvant chemotherapy.

### Treatment outcome and survival

The mean follow-up of the population was 21.9 months (2.1–100.9).

**Figure 1:**
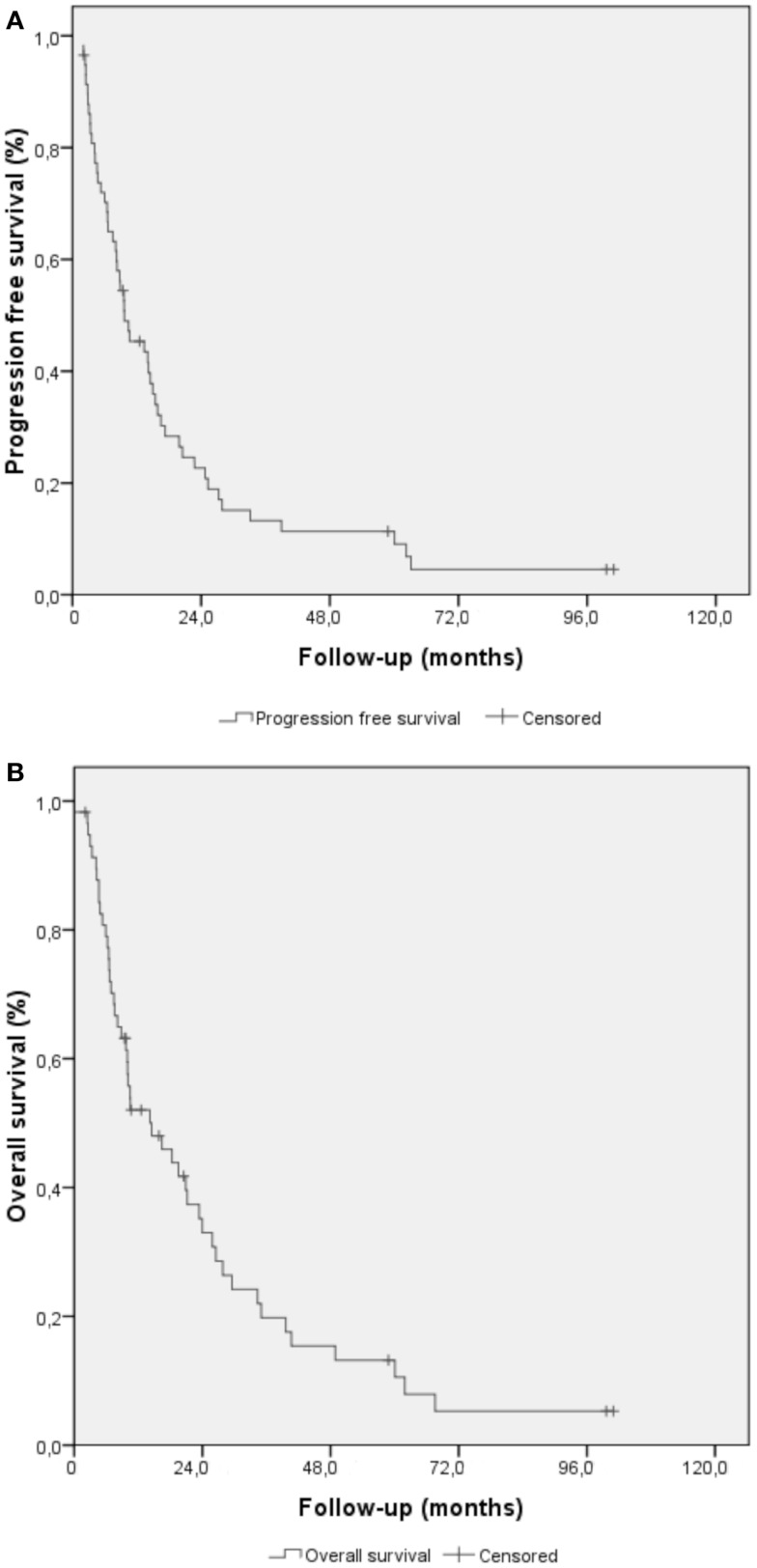
**Kaplan–Meier methods for elderly patients treated for esophageal and gastroesophageal cancer**. **(A)** Progression-free survival curve **(B)** overall survival curve.

The median PFS of the overall population was 9.6 ± 3.9 months (Figure 1A). Using univariate analysis, PS status and age <78 years (inferior to the median) were the only significant predictive factors for PFS (*p* = 0.012 and *p* = 0.019, respectively). The following factors were not significantly associated with PFS: sex, Charlson score, surgery, concomitant chemotherapy, and RT dose (see Table 3). Thirty-four patients relapsed: 17 loco-regionally, 8 with distant metastases, and 9 with both loco-regional and distant relapse. The 2- and 3-year-cumulative rates of local recurrence were 36.2 and 41.4%, respectively. The only positive predictive factor for local control was age: 2-years local relapse for patients <78 years was 64.0%, vs. 92.6% for patients ≥78 years (*p* = 0.021). Concerning metastatic recurrence, no prognostic factors were identified and the preferential localizations were mainly pleuropulmonar (*n* = 5), peritoneal (*n* = 3), hepatic (*n* = 2), and cerebral (*n* = 1), with a few unknown data (*n* = 5).

**Table 3:** **Univariate analysis for overall and progression-free survival in population treated in a curative intent**.

		Overall survival		Progression-free survival (24 months)	
	*N*°	Survival: *N*°(%)	*p*-Value	Survival: *N*°(%)	*p*-Value
**GENDER**					
Men	43	12(27.9)	0.65	9(20.9)	0.64
Women	15	3(20.0)		3(20.0)	
**AGE**					
<78 years	29	11(37.9)	0.048	10(34.5)	0.019
≥78 years	29	4(13.8)		2(6.9)	
**WHO PERFORMANCE STATUS**					
0	12	5(41.7)	<0.001	4(33.3)	0.012
1	38	10(26.3)		8(21.0)	
2	6	0(0)		0(0)	
3	2	0(0)		0(0)	
**CHARLSON SCORE**					
3–4	37	9(24.3)	0.80	7(18.9)	0.43
≥5	21	5(23.8)		5(23.8)	
**LOCALIZATION OF OC**					
Upper and medium	7	1(14.3)	0.27	1(14.3)	0.49
Low and GOJ	51	14(27.5)		10(19.6)	
**HISTOLOGY**					
SCC	14	2(14.3)	0.59	2(14.3)	0.42
Adenocarcinoma	43	13(30.2)		9(20.9)	
***T***					
*T*0/*T*1/*T*2	3	3(100)	0.67	1(33.3)	0.78
*T*3/*T*4	43	11(25.6)		11(25.6)	
***N***					
*N*0	5	2(40.0)	0.57	2(40.0)	0.76
*N* > 0	43	11(25.6)		10(23.2)	
**SURGERY**					
Yes	8	4(50.0)	0.035	3(37.5)	0.093
No	50	11(22.0)		9(18.0)	
**CONCOMITANT CHEMOTHERAPY**					
Yes	47	12(25.5)	0.31	14(29.8)	0.49
No	11	3(27.3)		3(27.3)	
**RADIATION DOSE**					
<50.4 Gy	27	8(29.6)	0.86	4(14.8)	0.93
≥50.4 Gy	31	7(22.6)		6(19.4)	

*OC, esophageal cancer; GOJ, gastroesophageal junction; SCC, squamous cell carcinoma*.

The median overall survival (OS) of the overall population was 14.5 ± 4.7 months (standard deviation), and 2 and 3 years-overall survival rates were 25.9 and 15.5%, respectively (Figure 1B). Using univariate analysis, the OS was positively influenced by age <78 years (*p* = 0.048, Figure S1 in Supplementary Material), a good PS (*p* < 0.001, Figure S2 in Supplementary Material), and the realization of surgery (*p* = 0.035). Details of the univariate analysis for OS are given in Table 3.

**Figure 2:**
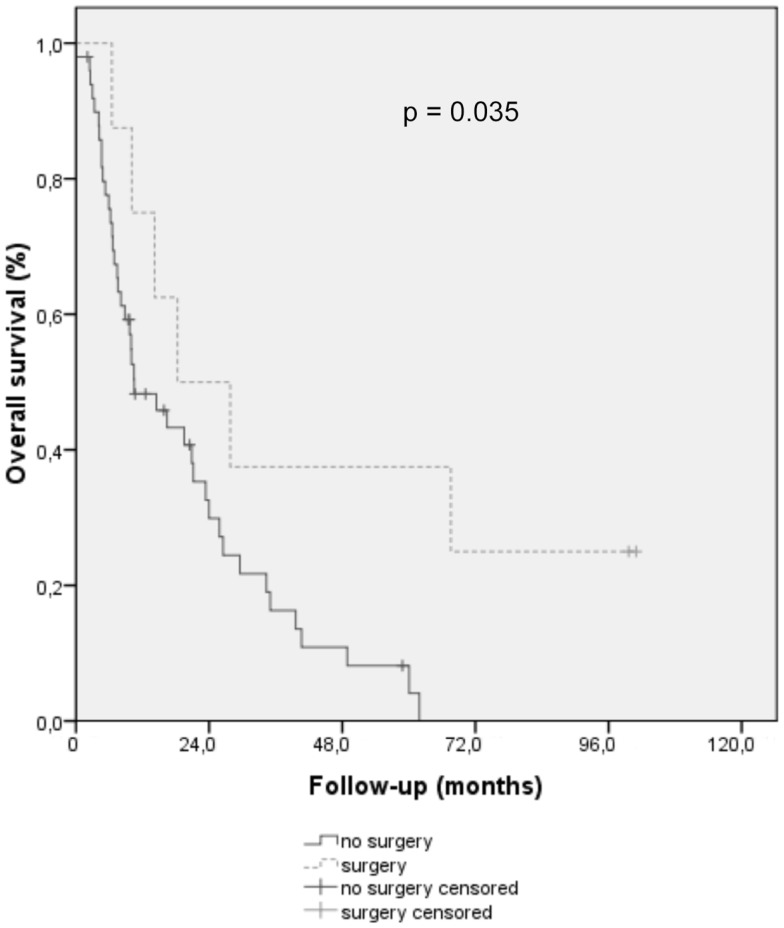
**Overall survival curve using Kaplan–Meier methods for elderly patients treated for esophageal and gastroesophageal cancer, with surgery (dotted line) or without (full line)**.

Curative esophagectomy (Figure 2) was associated with a median OS of 18.3 ± 9.7 vs. 10.5 ± 3.5 months for patient without surgery (*p* = 0.035).

There was no significant difference in terms of OS in univariate analysis for sex, Charlson score, histology, degree of differentiation, and tumor size >50 mm, usTN, pTN, RT dose, and concomitant chemotherapy. Accordingly with the univariate analysis results, none of the prognostic factors were significative in multivariate analysis.

47/58 died within 2 years: 30/47 deaths were due to OC disease progression, 6/47 due to complications of therapy, and the remainder to non-cancer (6/47) or unknown causes (5/47).

### Tolerance

During RT or RCT, grade 1–2 and 3–4 side effects occurred in 35 patients (60.3%) and in 18 patients (31%), respectively. The main hematologic side effects were febrile neutropenia (*n* = 7, 14.9%), thrombopenia (grade 3–4: *n* = 6, 12.8%), and anemia (grade 3–4: *n* = 3, 6.4%). The main non-hematologic side effects were dysphagia (grade 3–4, *n* = 9, 19.1%), nausea (grade 3–4: *n* = 3, 6.4%), and asthenia (grade 1–2: *n* = 6, 12.7% and grade 3–4: *n* = 1). There were only a few grade 1–2 mucositis and skin toxicity (*n* = 4 and *n* = 1, respectively). Interruptions of radiation therapy occurred in 13 patients (22%), temporarily (*n* = 7) or definitively (*n* = 6) at a mean dose of 35.8 Gy (26–40 Gy).

During RCT (*n* = 47), 18 patients (38.2%) had to be hospitalized, mainly for severe malnutrition (*n* = 7) or febrile neutropenia (*n* = 7). One toxic death occurred during RCT (asthenia, dysphagia).

Patients with exclusive RT (*n* = 11) had no hematologic side effects but more grade 3–4 asthenia (*n* = 5) and a comparable dysphagia (grade 3–4, *n* = 2, 18.2%) and hospitalization rate (*n* = 3, 27.3%). One toxic death occurred during exclusive RT (asthenia, severe malnutrition).

Surgical complications were described as follows: one gastric necrosis after gastroplasty, one anastomotic fistula with sub-cutaneous emphysema (both of them recovered after transfer in an intensive care unit), one anastomotic stenosis (2 months after the transthoracic procedure, treated by endoscopic dilatations), and one recurrent paralysis (with a good rehabilitation). None of the eight patients operated died in the 30 days after surgery.

## Discussion

We present here a large cohort of elderly patients treated with radiotherapy for an esophageal cancer.

Due to its retrospective nature, our analysis suffers from limitations, including the absence of a consistent onco-geriatric evaluation and systematic integration of quality-of-life assessment. Furthermore, a median follow-up of 21.9 months is rather short to precisely evaluate overall and/or specific survival, as well as their prognostic factors. This might explain why some validated prognostic factors do not reach significance in our results (e.g., T stage). In addition, therapeutic strategies were rather heterogeneous, in part due to the improvements made over the 10 years inclusion period. Finally, one could argue that SCC and adenocarcinoma are different diseases, even though most analysis including ours did not show any major differences in outcome.

On the other hand, this study concerned a consistent number of patients treated in only two experimented cancer centers, avoiding misleading factors such as variations of clinical staging between physicians. Technical reproducibility was guaranteed by the fact that the same team performed the endoscopic evaluation in the two centers.

In comparison with published data, our population is more homogeneous regarding age, as we chose an inferior limit at 75 years instead of 70 years (11) or 65 years (12). Furthermore, patients were not selected for PS or co-morbidity, contrary to the study of Anderson et al. in which only patients with Karnofsky Index >70% were included and in the study of Servagi-Vernat et al. in which 50% of patients were excluded for PS >2 or Charlson score >4. Consequently, our population can be considered as representative of routine clinical practice. Finally, every patient’s case was discussed in a multidisciplinary meeting with a medical oncologist, a radiation oncologist, and a surgeon. All the range of treatments, even surgery, could be discussed, which prevent the risk of undertreatment that is huge in elderly patients.

The results we found in terms of OS and PFS are consistent with those published in the literature, notably in the largest cohort of 109 elderly patients treated with exclusive RCT with a median OS and PFS of 15.2 and 8.3 months, respectively (16). In another small prospective study of 22 patients >75 years old treated by exclusive RCT (50 Gy and weekly cisplatin), the median OS was 15 months (11). Only a study by a team from Memorial Sloan Kettering described a better OS (median of 35 months) but with only 22 patients included, who were younger and with fewer comorbidities compared to our study (12).

Moreover, patient outcomes described here are not so far from the results published in the literature for younger counterparts. In the most important clinical trials, including patients below the age of 75 years old, median OS ranged between 13.0 and 19.3 months (5, 8, 17). This emphasizes the fact that curative treatments of OC should not be rejected only on age’s argument.

In our study, the best results in terms of OS were obtained for patients who underwent neoadjuvant RCT followed by esophagectomy. Coia et al. also found a superiority of esophagectomy in the 2 years survival rate (50.2 vs. 31.2%) in the general population receiving RT in MSKCC between 1992 and 1994 (18). In a study by Ruol and colleagues, concerning 62 patients over 80 years old, esophagectomy was found to be a prognostic factor in OC with an important difference in OS: 14.6 vs. 5.1 months in resected and non-resected patients, respectively (19). Despite the fact that esophagectomy is a major surgical procedure, Markar et al. indicated that there were no significant differences in terms of length of hospitalization and survival rate for patients over 80 years in comparison to younger counterparts (20). In our study, surgery was associated with local complications (anastomosis stenosis, necrosis of gastroplasty, and fistula) but without perioperative death. Along this line, previous results showed that a lower 30-day mortality rate after esophagectomy is obtained when surgery is realized in a specialized center (21). Together with previous evidence (22), our results suggest that surgical resection is feasible even in elderly patients, which was not predictable, because age still appears as a risk factor in certain predictive scores (23).

Concerning RCT tolerance, our results are close to the data of the literature as Tougeron and Anderson presented 22 and 36% of grade 3–4 toxicity, respectively. In elderly patients with 5 FU or platinum regimen, febrile neutropenia is a major issue that must be carefully managed, notably relying on GCSF (24) or eventually with an antibiotic prophylaxis. Another challenge is to keep a calories intake around 1500 kcal, as far as possible using nutritional supplements or enteral nutrition with gastrostomy or jejunostomy (25). Onco-geriatric systematic evaluation is another clue to decrease toxicity by managing age-related fragility (26–28). We can also hypothesize that toxicity of RT will decline in the next few years thanks to the technical progress of RT as gating technique and new treatment planning techniques as intensity-modulated radiotherapy including volumetric modulated arc therapy (29–31).

In conclusion, our results suggest that a curative treatment of OC based on RT is feasible on patient over 75 years, with an acceptable tolerance. The outcome remains poor but consistent with results in younger patients. With the use of a comprehensive geriatric assessment, no treatment modification is needed whatever the age of patients. Surgical resection should be recommended as the standard treatment for adenocarcinoma of lower esophagus or gastroesophageal junction in elderly patients with good PS and low co-morbidity profile, as it is in younger patients.

## Conflict of Interest Statement

The authors declare that the research was conducted in the absence of any commercial or financial relationships that could be construed as a potential conflict of interest.

## Supplementary Material

The Supplementary Material for this article can be found online at http://www.frontiersin.org/journal/10.3389/fonc.2014.00100/abstract

Figure S1 **Overall survival curve using Kaplan–Meier methods for elderly patients treated for esophageal and gastroesophageal cancer, with age <78 years (blue line) or >78 years (green line)**.

Figure S2 **Overall survival curve using Kaplan Meier methods for elderly patients treated for esophageal and gastroesophageal cancer according performance status (PS)**.

Table S1 **Pathological findings after oesophagectomy**.
