# Supplementary figures and images for: Radio(chemo)therapy in Elderly Patients with Esophageal Cancer: A Feasible Treatment with an Outcome Consistent with Younger Patients
Source: Front Oncol. 2014 May 12;4:100. doi: 10.3389/fonc.2014.00100 (PMC4026749; doi:10.3389/fonc.2014.00100)

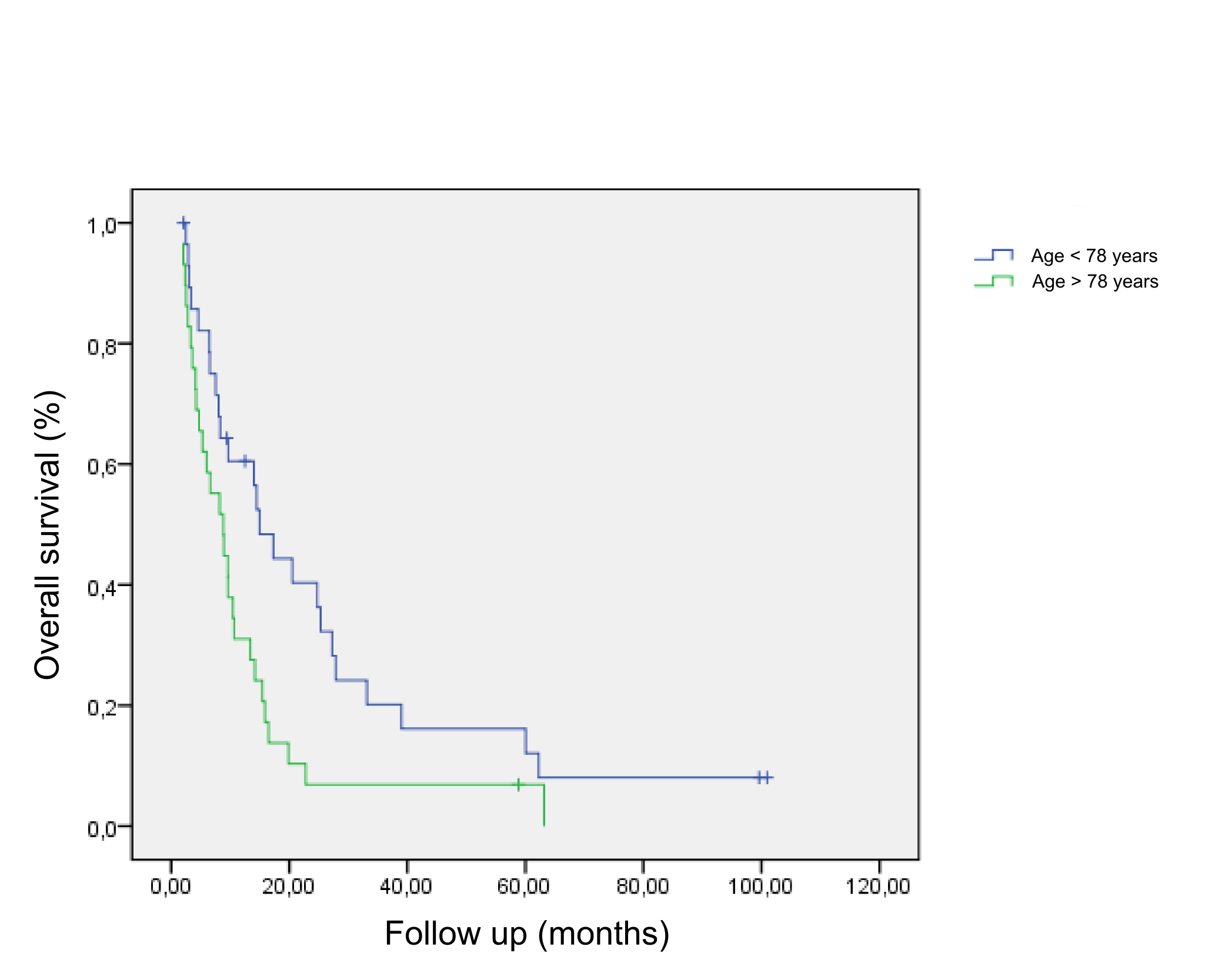

Supplement: Figure S1 — Overall survival curve using Kaplan–Meier methods for elderly patients treated for esophageal and gastroesophageal cancer, with age <78 years (blue line) or >78 years (green line). [file Presentation1.ZIP › Figure S1.tif]

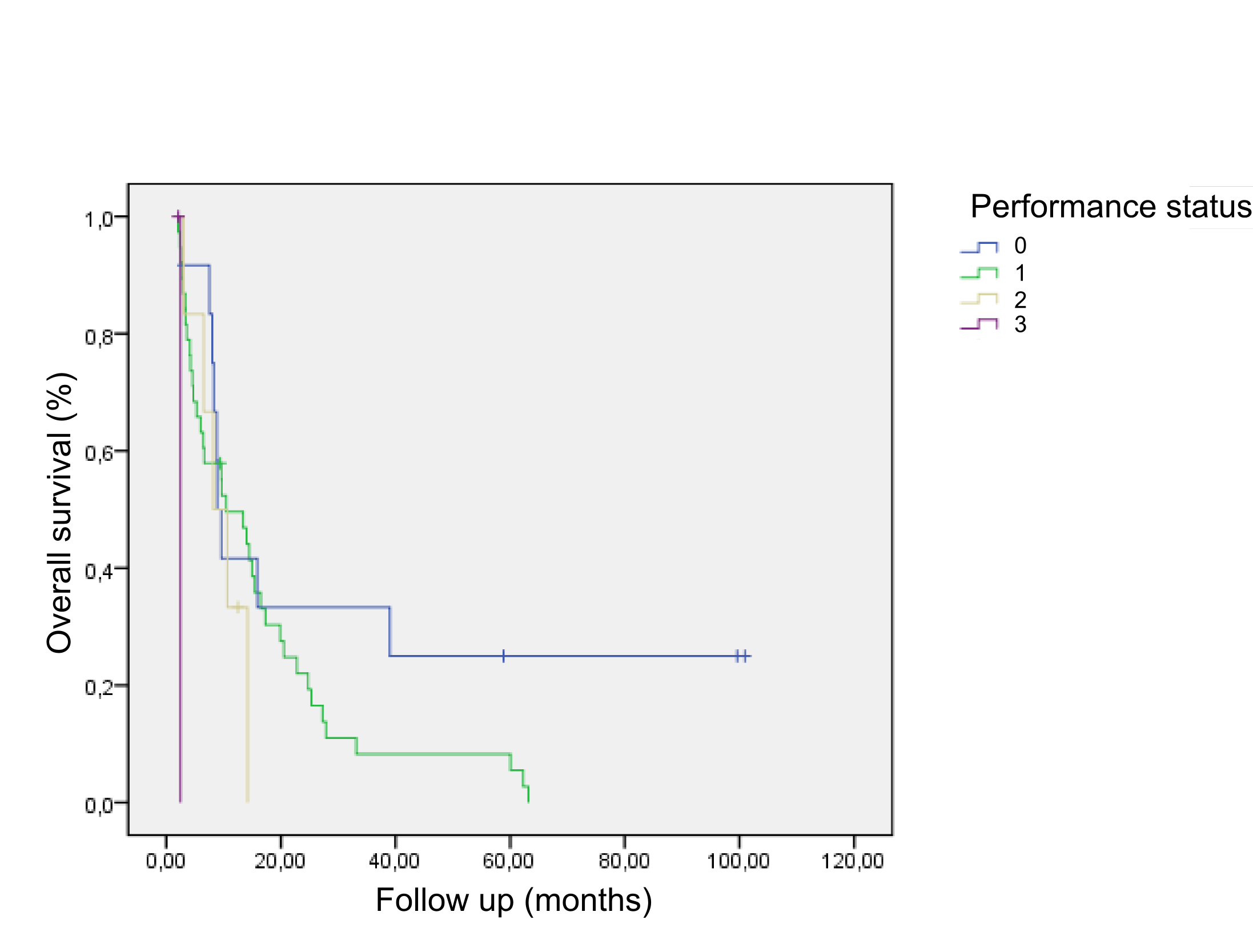

Supplement: Figure S1 — Overall survival curve using Kaplan–Meier methods for elderly patients treated for esophageal and gastroesophageal cancer, with age <78 years (blue line) or >78 years (green line). [file Presentation1.ZIP › Figure S2.tif]
